# New Insights on Iron-Trimesate MOFs for Inorganic As(III) and As(V) Adsorption from Aqueous Media

**DOI:** 10.3390/nano15010036

**Published:** 2024-12-29

**Authors:** Afef Azri, Marwa Ben Amar, Khaled Walha, Clàudia Fontàs, José Elías Conde-González, Victoria Salvadó, Eladia M. Peña-Méndez

**Affiliations:** 1 Department de Química, Facultat de Ciències, Universitat de Girona, C/Mª Aurèlia Capmany, 69, 17003 Girona, Spain; afef.azri@udl.cat (A.A.); marwa.ben@imb-cnm.csic.es (M.B.A.); claudia.fontas@udg.edu (C.F.); 2 Laboratory of Material Sciences and Environment, Faculty of Sciences, University of Sfax, Route de la Soukra Km 3.5, BP 1171, Sfax 3000, Tunisia; khaled.walha@fss.rnu.tn; 3 Unidad Departamental de Química Analítica, Departamento de Química, Facultad de Ciencias, Universidad de La Laguna, Avda. Astrofísico Fco. Sánchez, s/n, 38206 La Laguna, Spain; jconde@ull.edu.es

**Keywords:** inorganic arsenic species, iron-trimesate MOFs, adsorption, porosity

## Abstract

Arsenic contamination of water endangers the health of millions of people worldwide, affecting certain countries and regions with especial severity. Interest in the use of Fe-based metal organic frameworks (MOFs) to remove inorganic arsenic species has increased due to their stability and adsorptive properties. In this study, the performance of a synthesized Nano-{Fe-BTC} MOF, containing iron oxide octahedral chains connected by trimesic acid linkers, in adsorbing As(III) and As(V) species was investigated and compared with commercial Basolite^®^F300 MOF. Despite their similarities in composition, they exhibit distinct structural characteristics in their porosity, pore size, and surface areas, which affected the adsorption processes. The kinetic data of the adsorption of As(III) and As(V) by both Fe-MOFs fitted the pseudo second-order model well, with the kinetic constant being higher for Basolite^®^F300 given its higher porosity. Intraparticle diffusion was, in both cases, the rate controlling step with the contribution of film diffusion in the adsorption processes, which achieved equilibrium after 1 h. The maximum adsorption capacity for As(V), 41.66 mg g^−1^, was obtained with Basolite^®^F300 at the 6.5–10 pH range, whereas Nano-{Fe-BTC} showed a different behaviour as maximum adsorption (14.99 mg g^−1^) was obtained at pH 2. However, both adsorbents exhibited the same performance for As(III) adsorption, which is not adsorbed at pH < 9. The Langmuir adsorption isotherm model fitted well for As(III) and As(V) adsorption by Nano-{Fe-BTC} and As(III) by Basolite^®^F300, whereas the Freundlich model fitted best for As(V) given its superior structural properties.

## 1. Introduction

Arsenic, which is classified as a metalloid, is the 20th most abundant element in the Earth’s crust and is a component of more than 250 minerals [1]. High concentrations of arsenic are stored in soil and water supplies through different pathways due to the geological conditions and anthropogenic activity [1,2]. The toxicity and non-biodegradability of this element cause water contamination that affects millions of people worldwide. Long-term exposure to arsenic and its ingestion, even at small concentrations, can lead to skin and lung cancer, neurological diseases, and diabetic disorders, and arsenic is listed as a first-group carcinogen by the International Agency for Research on Cancer (IARC) [3,4]. The World Health Organisation (WHO) and the US Environmental Protection Agency (USEPA) have established limits of 10 µg/L in drinking water to prevent people facing long-term chronic exposure to arsenic [5,6]. Arsenic toxicity is closely related to its oxidation state: the most common forms of arsenic in natural water are the inorganic forms of arsenate As(V) and arsenite As(III), which is 60 times more toxic than As(V) [7]. As(III) is always more mobile and most commonly occurs as the uncharged arsenous acid H_3_AsO_3_, making its removal more difficult by anion exchange and coagulation. As a result, a pretreatment with oxidants to convert As(III) to As(V) is required in order to remove As(III) efficiently [8].

Several studies have reported that, in many regions across the world, arsenic concentrations exceed the recommended WHO values for drinking water [9,10]. Given this situation, it is important to develop methods for the removal of arsenic that are easy to apply and highly efficient. Physicochemical methods including oxidation, precipitation, ion exchange, separation (reverse osmosis, nanofiltration, and electrodialysis), electrocoagulation, and adsorption have been developed for this purpose. Of these, adsorption is currently considered the most recommended method, since it is simple, efficient, eco-friendly, and economically affordable [11].

A number of sorbents, including activated carbons, activated alumina, zeolites, clay, minerals, natural and synthetic oxides, biochars, and biosorbents, have already been tested for their capacity to remove arsenic from aqueous media [12,13]. However, their usefulness is limited due to poor performance and their lack of selectivity [14,15]. Research into nanomaterials has led to the development of nanoparticles, which, due to their high specific surface area, high reactivity, and high specificity, are emerging as interesting potential alternatives to existing conventional sorbents. When iron-based nanoparticles, such as zero-valent iron nanoparticles (nZVI) and iron oxide nanoparticles (Fe_3_O_4_ and γ-Fe_2_O_3_), have been used as nanosorbents, they have been found capable of removing arsenic with up to 10 times their micron size [14,16,17,18].

Metal organic frameworks (MOFs) are a new class of porous crystalline hybrid materials formed by metal nodes and multidentate organic ligands [19,20]. They present two major advantages in adsorption applications over other nanoparticles: firstly, the presence of coordinative unsaturated sites (CUS) or open metal sites in their structure make them readily accessible, and, secondly, MOFs have high thermal and mechanical stability, which allow them to withstand common aggregation problems. These advantages, together with their relatively simple and easy synthesis, high surface areas, and nanoscale pore sizes and shape, lead to better performance in removing heavy metals and metalloids from water than other porous adsorbents [21,22]. Several studies have analysed the ability of MOFs in removing both As(V) and As(III) [20,23,24,25]. Among these, iron-based MOFs have exhibited a great affinity for As(V) and their capacities and kinetics have been improved from MIL-53 to MIL-101 [26,27,28]. Iron and 1,3,5benzenetricarboxylic (Fe-BTC) metal-organic coordination polymers have been synthesised via a simple solvothermal method and applied to the adsorption of As(V) species from water solutions [29]. Moreover, the preparation of MOFs with trivalent octahedral metals and simple aromatic carboxylates, such as trimesate, has resulted in MOFs that are highly stable with hierarchical mesopores. However, certain synthetic routes of the Fe-BTC MOFs can cause defects in their structure that can improve their sorption properties [30]. In the case of ZIF-8, its capacity was increased by preparing composite ZIF-8 with metal oxides (e.g., Fe_3_O_4_@ZIF-8 and MnO_2_@ZIF-8) [31]. The aim of this study is to characterise the kinetics and the adsorption processes of As(V) and As(III) by using two iron-based porous MOFs, synthesised Nano-{Fe-BTC} and commercial Basolite^®^F300. These MOFs have a similar chemical composition and differ in their structural characteristics. The role of porosity, pore size, and surface area of iron-trimesate MOFs on the mechanism and performance of the adsorption processes has been investigated, as well as the effect on the adsorption efficiency of parameters such as contact time, pH, the amount of adsorbent, and the initial arsenic concentration.

## 2. Materials and Methods

### 2.1. Materials

Basolite^®^F300 (Sigma-Aldrich, Chemie Gmbh, Steinheim, Germany) has a hybrid super tetrahedral build-up from an octahedron of oxo-centred trimmers of iron(III) that are connected by trimesate anions [30]. The synthesis of Nano-{Fe-BTC}, a Basolite-like MOF, was performed following the principles of green chemistry and has been described in a previous article [32]. The elemental surface composition of both sorbents was determined by EDS, their morphologies by SEM, and their thermal stability by TGA. Moreover, the specific surface areas, pore size distribution, and pore volumes were measured by the Brunauer, Emmett, and Teller method (BET). The morphology and size of the synthesised materials were examined using transmission electron microscopy (TEM) (JEOL 2010, Tokyo, Japan). A scanning electron microscope (JEOL JSM 6300, Tokyo, Japan) with a resolution of 3.5 nm combined with an energy-dispersive X-ray spectrometer (Oxford 6699 ATW, Oxford, Oxfordshire, UK) was used to determine the surface composition of the materials. Powder X-ray diffraction (XRD) data were collected at 298.2 K using a D8 Advance instrument (Bruker AXS GmbH, Karlsruhe, Germany) equipped with CuKα radiation and a curved graphite monochromator on the diffracted beam. Data were collected in the 5–35° 2θ range with an 0.05° step width.

FTIR analyses of both sorbents before and after As(III) and As(V) sorption were carried out in the 600–3600 cm^−1^ region using an Agilent Cary 630 FTIR spectrometer (Agilent, Santa Clara, CA, USA) equipped with a diamond attenuated total reflectance (ATR) accessory.

Stock solutions of 1000 mg L^−1^ of As(III) and As(V) were prepared by adding appropriate amounts of NaAsO_2_ and Na_2_HAsO_4_·7H_2_O (Panreac, Barcelona, Spain) in doubly deionised water (MilliQ) obtained from a Millipore water purification system (18.2 MΩ cm^−1^, Millipore, MA, USA).

Arsenic samples were analysed by either ICP-AES (Agilent 4200 MP-AES, Agilent Technologies, Santa Clara, CA, USA) or ICP-MS (Agilent 7500c Agilent Technologies, Santa Clara, CA, USA), depending on the arsenic concentrations. Calibration curves were plotted by analysing six individual arsenic standard solutions prepared at the same pH and matrix media of the experimental solutions by dilution of 1000 mg L^−1^ standard solution (SPEX CertiPrep, Stanmore, UK).

The pH of the working solutions (pH_i_) was adjusted to the desired value by adding HNO_3_ 0.1 M and NaOH 0.1 M. NaOH (98%, Panreac, Barcelona, Spain) and 65% HNO_3_ (Merck, Darmstadt, Germany). The pH of the solutions was measured using a Basic 20+ pH-meter (Crison Instruments, S.A., Allela, Spain), which was previously calibrated with buffer solutions of pH 7.0 and pH 4.0.

### 2.2. Batch Experiments

Batch studies were carried out with each sorbent and As(III) and As(V) solutions to characterise the adsorption process under controlled conditions. The first experiments were performed at different contact times in order to determine the kinetics of the sorption processes and the equilibrium time. The effect of the amount of sorbent, pH of the aqueous metalloid solution, and initial metalloid concentration was studied by varying the corresponding parameters and maintaining the other experimental conditions.

The tests were conducted at a constant room temperature of 22 ± 1 °C. In each series of tests, 20 mL of the arsenic solution (As(III) or As(V)) with concentrations ranging from 4 to 25 mg L^−1^ were introduced into 25 mL glass extraction tubes containing precisely weighted amounts of the selected sorbent. The mixture was then agitated using a rotary mixer (Dinko Instruments, Barcelona, Spain) until the chemical reaction reached equilibrium. Agitation velocities were 20 rpm or 50 rpm with Basolite^®^F300 and 20 rpm for Nano-{Fe-BTC}. The sorbent and the solution were separated by centrifugation and filtration using 0.2 µm syringe filters before determining the remaining arsenic concentrations in the filtrate.

The arsenic uptake rate in each experiment and the amount of adsorbed arsenic at the equilibrium q_e_ (mg g^−1^) were calculated using Equation (1) and Equation (2), respectively:

(1) $$Adsorption\;\left(\%\right)=\frac{\left(C_{i}-C_{eq}\right)}{C_{i}}\cdot 100$$

where C_i_ and C_eq_ (mg L^−1^) are the concentrations of arsenic at the initial and equilibrium times, respectively:

(2) $$q_{e}=\frac{\left(\left(C_{i}-C_{eq}\right)\cdot V\right)}{W}$$

where V (L) is the volume of the solution, and W (g) is the mass of dry adsorbent used.

Then, 2 mg of Basolite^®^F300 or Nano-{Fe-BTC} were added to 20 mL of a 10 mg L^−1^ As(V) or As(III) solutions to study the kinetics of the sorption process. Preliminary tests were performed to determine the optimum pH of the arsenic solutions to achieve the maximum adsorption efficiency by both adsorbents. This pH was 11 for As(III), whereas in the case of As(V) the pH was 2 for Nano-{Fe-BTC} and 6 for Basolite^®^F300. Contact time was up to 90 min, and agitation was at 20 rpm and 50 rpm for Basolite^®^F300, and at 20 rpm for Nano-{Fe-BTC}. For the latter, it was not possible to perform the kinetic tests at 50 rpm given that equilibrium was achieved at t < 1 min at this agitation velocity. The kinetic tests led to an equilibrium time of 1 h being established.

The effect of the pH of the aqueous arsenic solutions on the adsorption efficiency was studied at different initial pH values (pH_i_ = 2, 4, 6, and 9) for As(V) and (pH_i_ = 2, 7, 9, and 11) for As(III), whilst the other parameters were maintained.

The effect of the amount of the Fe-BTC adsorbents on the adsorption capacity was investigated by adding amounts of 2 and 10 mg of Basolite^®^F300 to a 10 mg L^−1^ As(V) solution at pH 6 and to 10 mg L^−1^ As(III) solution at pH 11. The same conditions were used in the experiments with Nano-{Fe-BTC}, except that, in the case of As(V), the pH of the solution was 2.

The effect of As(III) and As(V) concentrations on the adsorption capacity was studied by varying these concentrations from 4 to 25 mg L^−1^ with 10 mg of adsorbent. The pH was adjusted to 11 in the case of As(III) solutions and 2 in the case of As(V) solutions when Nano-{Fe-BTC} was the adsorbent and to pH 6 when the adsorbent was Basolite^®^F300. Batch experiments were also performed with Basolite^®^F300 using lower concentrations (0.5 and 1 mg L^−1^ of As(V) at pH 6). The agitation time was 1 h.

## 3. Results and Discussion

### 3.1. Characterisation of Basolite^®^F300 and Nano-{Fe-BTC}

The morphology of the synthesised Nano-{Fe-BTC} MOF was examined by transmission electron microscopy at different magnifications (Figure S1), proving the semi-crystallinity of the structure and the octahedral shape of this MOF, as well as showing the interconnection between the nanoporous particles. As can be observed in Figure S1, the synthesised Nano-{Fe-BTC} MOF is more homogeneous than the Basolite^®^F300 nanoparticles that agglomerate in the bulk state.

**Figure 1:**
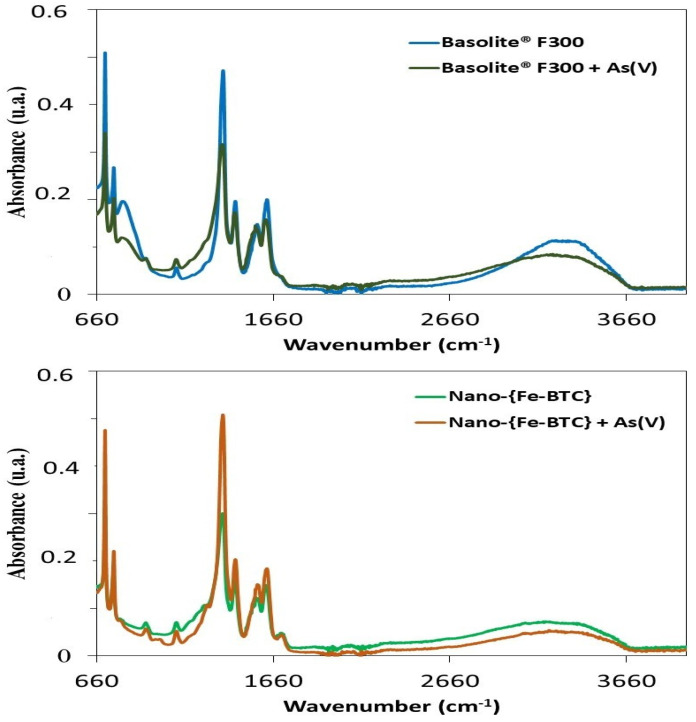
FTIR spectra of Basolite^®^F300, As(V)-loaded Basolite^®^F300, Nano-{Fe-BTC}, and As(V)-loaded Nano-{Fe-BTC}.

The elemental analysis of the adsorbent surface performed by EDX showed that Basolite^®^F300 is composed of carbon (74.39%), oxygen (21.84%), and iron (2.97%), whereas Nano-{Fe-BTC} MOF is composed of a similar percentage of carbon (78.05%), slightly less oxygen (17.09%), a greater percentage of iron (3.68%), and chlorine (0.87%). EDX results and those of XRD and TGA show that both sorbents have similar characteristics and stability [32]. The surface area and pore volume of these nanocrystallines were measured by the BET method, finding 840 m^2^ g^−1^ and 0.22 cm^3^ g^−1^ for Basolite^®^F300 [33] and 427.2 m^2^ g^−1^ and 0.53 cm^3^ g^−1^ for Nano-{Fe-BTC}. Porosity, determined by N_2_ adsorption–desorption isotherms at 77 K, is higher for Basolite^®^F300 (29.95%) than Nano-{Fe-BTC} (10.12%), and these results suggest the presence of mesopores with a diameter in the upper size range (between 2 nm and 50 nm) in the case of Nano-{Fe-BTC} MOFs [32]. FTIR analyses were 194 performed before and after adsorption with the two Fe-BTC MOFs. The FTIR spectra of Basolite^®^F300 and Nano-{Fe-BTC} MOFs have similar profiles (Figure 1) with bands in the 650–1800 cm^−1^ region, which is generally considered to be a fingerprint of MOFs. However, differences in intensity between the two materials are found in the 950–1300 cm^−1^ region. The peaks obtained at 1629 cm^−1^, 1454 cm^−1^, and 1380 cm^−1^ are due to the presence of asymmetric and symmetric stretching vibration of carboxylate groups of the BTC ligand. The peak registered at 762 cm^−1^ suggests the presence of Fe-O, indicating the coordination of BTC to the iron sites. Moreover, the synthesised Fe-BTC presents a broad band at 3197 cm^−1^, corresponding to water and OH of the material, and a band at 2143 cm^−1^, which is assigned to a stretching vibration of -C=C-.

### 3.2. Characterisation of the Sorption Processes

In order to elucidate the adsorption mechanism, characteristics of the materials before and after the adsorption of arsenic by energy-dispersive spectroscopy (EDS), X-ray powder diffraction (XRD), and infrared spectroscopy (IR) were carried out. Moreover, different kinetic and isotherm models were applied to characterise the adsorption processes. Through EDS analysis, it was determined that both Basolite^®^F300 and Nano-{Fe-BTC} MOFs exhibit proficient arsenate adsorption capabilities. Arsenic was detected in the MOFs loaded with arsenate aqueous solution (Figure S2). In the literature, X-ray photoelectron spectroscopy (XPS) was conducted to further elucidate the adsorption mechanism and the interaction between As(V) and MOF materials. Zhu et al. reported that As(V) is adsorbed within the interior (pore) of the Fe-BTC polymer rather than remaining on the outer surface, which is seen by the absence of any arsenic signal in the XPS spectrum after adsorption. This result was confirmed by TEM mapping of the Fe-BTC polymer [29]. However, a strong new Fe-O-As peak was detected by XPS using the MIL-101(Fe), highlighting the contribution of the coordination between As and Fe to the adsorption of As(V) by these MOFs, which have a higher degree of crystallinity and a higher percentage of mesopores (55.44%) than Basolite^®^F300 and Nano-{Fe-BTC} [34].

The XRD spectra of Basolite^®^F300 reveal that, after the adsorption of As(V), the intensities of the diffraction lines did not change significantly, showing no affectation of the crystalline nature of MOFs, as seen in Figure S3.

Similar results were obtained for the adsorption of As(III) using Nano-{Fe-BTC} as the adsorbent. The semi-amorphous nature of these MOFs complements the adsorption properties, providing defect sites for As adsorption [32,35]. Similar findings were obtained using Zn-MOF-74, demonstrating the high stability of these materials after exposure to arsenate [36]. Berardozzi et al. also found that the adsorption of arsenic into MIL-100(Fe) did not cause changes to the XRD patterns [37]. These results contrast with findings for other MOFs used in studies by other groups in which the diffusion of arsenate was found to destroy the mesopores of MIL-100(Fe), MIL-100(Al), and MIL-101(Fe) [27,34,38]. Consequently, depending on the amount of arsenic loaded into the MOFs and the degree of crystallinity of the material, the coordination between the metal cluster and the linkers within an MOF structure could either be disrupted or unaffected.

To investigate the interaction of these materials with different inorganic arsenic species, IR analyses were performed with both Fe-BTC MOFs alone as well as loaded with As(III) and As(V). As shown in the FTIR spectra in Figure 1, the intensity of the peaks clearly increased after the adsorption of As(V). In addition, the appearance of new peaks between 800 and 900 cm^−1^ in the MOFs loaded with As(V) spectra can be attributed to the Fe–O–As coordination. In a similar study, a band at 825 cm^−1^, attributed to Fe–O–As, was observed in the FTIR spectra, confirming the interaction of As(V) with Fe(III) inside the MOFs [27]. In the FTIR recorded in our laboratory, this peak cannot be clearly distinguished, but a band can be observed in this region, whose intensity is higher for Basolite^®^F300 loaded with As(V) than for Nano-{Fe-BTC} loaded with As(V). Similar results were obtained when the FTIR spectra were recorded with both Basolite^®^F300 and Nano-{Fe-BTC} loaded with As(III) at pH 11. To gain further insights into the adsorption mechanisms and to determine the uptake rate of inorganic arsenic species (arsenite and arsenate) by Basolite^®^F300 and Nano-{FeBTC} MOFs, kinetic and isotherm studies were performed. The effect of different parameters, such as the contact time, pH, amount of adsorbent, and initial concentration on the adsorption of arsenite and arsenate by Basolite^®^F300 and Nano-{Fe-BTC} MOF, was also investigated.

### 3.3. Kinetics of the Sorption Processes

Adsorption kinetics was investigated by using the pseudo-first-order (PFO) and the pseudo-second-order (PSO), as well as the intraparticle diffusion and Weber and Morris models to understand the rate-limiting mass transfer mechanisms [39]. Moreover, the Elovich model, which allows the mass and surface diffusion, and the activation and deactivation energy of the sorption system to be predicted, was applied to understand the nature of the adsorption processes (cf. for equations see Table S1).

**Figure 2:**
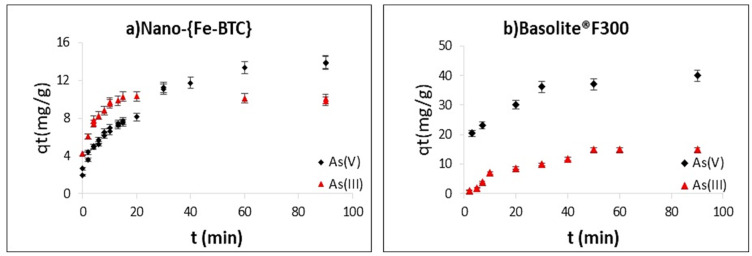
Effect of the contact time on the adsorption of As(III) and As(V): (**a**) 10 mg L^−1^ of As and 0.5 g L^−1^ of Nano-{Fe-BTC} at 20 rpm (As(V) at pH_i_ = 2 and As(III) at pH_i_ = 11); and (**b**) 10 mg L^−1^ of As and 0.1 g L^−1^ of Basolite^®^F300 at 50 rpm (As(V) at pH_i_ = 6.5 and As(III) at pH_i_ = 11) (n = 2).

The adsorption capacities (q_t_) at the different times for both arsenic species are shown in Figure 2. As can be seen, equilibrium was reached after approximately 1 h. The initial pH (pH_i_), of the aqueous solution for studying the adsorption of inorganic arsenic species by the two MOF adsorbents was optimised. For Basolite^®^F300, the adsorption capacity for As(V) increased pH_i_ values from 2 to 6.5 and then remained constant until pH_i_ = 10.5, whereas, for Nano-{Fe-BTC}, the maximum efficiency occurred at pH_i_ = 2. As for As(III) solutions, the pH_i_ was adjusted to 11. It was observed that, given that experimental conditions, the pH_i_ of the As(III) and As(V) solutions always decreased when they were placed in contact with the sorbents (Figure S5), affected by the acidity of these Fe-BTC MOFs. Hence, the pH at equilibrium was measured in all the tests.

**Figure 3:**
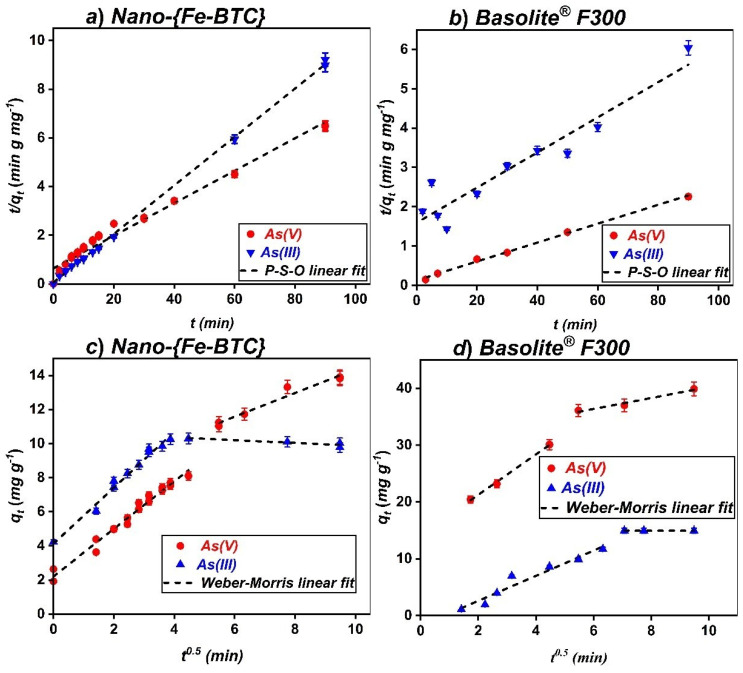
(**a**) PSO linear fit of the uptake of As (10 mg L^−1^) by Nano-{Fe-BTC} (0.5 g L^−1^) at pH_i_ = 2 for As(V) and pH_i_ = 11 for As(III), velocity (20 rpm); (**b**) PSO linear fit of the uptake of As (10 mg L^−1^) by Basolite^®^F300 (0.1 g L^−1^ at pH_i_ = 7 for As(V) and pH_i_ = 11 for As(III)), velocity (50 rpm); (**c**) WeberMorris linear fit of the uptake of arsenic species (10 mg L^−1^) by Nano-{Fe-BTC} (0.5 g L^−1^) at pH_i_ = 2 for As(V) and pH_i_ = 11 for As(III), velocity (20 rpm); and (**d**) Weber–Morris linear fit of the uptake of As (10 mg L^−1^) by Basolite^®^F300 (0.1 g L^−1^) at pHi = 7 for As(V) and pH_i_ = 11 for As(III)), velocity (50 rpm), (n = 2).

The kinetic parameters calculated from the application of the kinetic models (Table S1) are presented in Table 1. The determination coefficient R^2^ of the PSO model was ≥0.999 and the corresponding adsorption capacities were close to the experimental values for both arsenic species (III and V) and both MOFs (Figure 3a,b). Therefore, the experimental data fitting with the PSO model assumes that the adsorption process is mainly dominated by chemical adsorption controlled by the surface availability. The adsorption capacities for As(V) at the same velocity (20 rpm) are 12.2 mg g^−1^ and 14.9 mg g^−1^ for Basolite^®^F300 and Nano-{Fe-BTC}, respectively. However, the kinetic constant (k_2_) is ten times higher for Basolite^®^F300 given its higher porosity (29.95% vs. 10.12%) [32]. When an agitation velocity of 50 rpm was used with Basolite^®^F300, adsorption capacities of 41.7 and 22.3 mg·g^−1^ were obtained by As(V) and As(III), respectively.

The application of the Elovich and Weber–Morris models showed that two mechanisms are involved in the adsorption process, suggesting either the existence of two different interactions occurring between the adsorbent and adsorbate or the presence of two processes (chemisorption and ion exchange). As can be seen in Table 1, the determination coefficients are high for the Elovich model in the two steps (M1 and M2), indicating that the adsorption process involves a chemisorption step followed by a diffusion step. However, this second step is not clearly distinguished for As(III) adsorption. In order to elucidate the diffusion process, the Weber–Morris model (W-M) was applied (Figure 3c,d), revealing two steps corresponding to two different adsorption phases. The first one, at early adsorption times characterised by fast kinetics (K_WM1_), is associated with an intra-particle diffusion of either As(III) or As(V) from the solution to the pores inside the two MOFs. For the second step, at longer adsorption times (t^0.5^ ≅ 5 min^0.5^), where the kinetic constants K_WM2_ are much lower than K_WM1_ for As(V) for both MOFs, this corresponds to the adsorption that takes place once the sorbent is enriched by arsenic species. As can be seen in Figure 3c,d, it is linear in the case of As(III) for both Fe-BTC MOFs, indicating the equilibrium state of the system. However, there is a slight decrease in the case of As(V) and Nano-{Fe-BTC}, which indicates a decrease in the adsorption rate. In none of the cases did the fitted lines pass through the origin, showing that intraparticle diffusion is not the sole rate-limiting step of the adsorption process, suggesting that the boundary layer diffusion also controls the adsorption process and that the arsenic species diffuses through a stagnant film or boundary layer surrounding the particles of the adsorbents. The relatively high determination coefficients obtained by the application of the Liquid Film Diffusion model (LF-D) supported the latter assumption by demonstrating the existence of more than one mechanism and suggesting that three steps are involved in the adsorption of As(III) and As(V) by Basolite^®^F300 and the Nano-{Fe-BTC}: (i) the mass transfer across the external boundary layer of the adsorbent (liquid-film diffusion), (ii) the adsorption, and (iii) the diffusion through the pores of the MOFs.

### 3.4. The Effect of pH

The pH controls the adsorption of As(III) and As(V) as their speciation depends significantly on both the pH and the redox potential (Figure S4). Moreover, the pH of the zero-point charge (pH_pzc_) of the adsorbent is used to explain whether the adsorption mechanism is electrostatic or follows another mechanism, and to understand the effect of the pH on the adsorption process [40]. The pHpzc was determined by measuring the pH of a Nano-{Fe-BTC} MOF suspension in water and was found to be 4, whereas, in the case of Basolite^®^F300, it was 3.8 [32]. Given that the acidity of these Fe-BTC MOFs, the pH_i_ of the As(III) and As(V) solutions always decreased when they were placed in contact with the sorbents (Figure S5). Hence, the pH at equilibrium was measured in all the tests.

**Figure 4:**
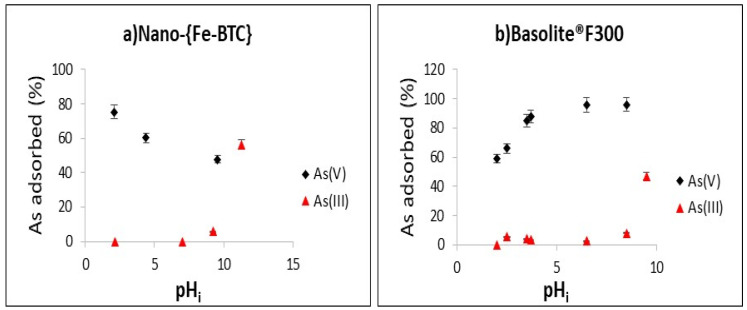
Effect of the initial pH_i_ on the adsorption of (**a**) 10 mg L^−1^ As by 0.5 g L^−1^ of Nano-{Fe-BTC} and (**b**) 5 mg L^−1^ of As by 0.5 g L^−1^ Basolite^®^F300, t = 1 h (n = 2).

The adsorption of As(III) and As(V) by Nano-{Fe-BTC} as a function of pH is depicted 338 in Figure 4a. The Nano-{Fe-BTC} showed its maximum adsorption capacity for As(V) at 339 pH 2 and further increases in the pH value led to a reduction in efficiency. At pH 2, H_3_AsO_4_ is the main species of As(V) (60%) (Figure S4), whereas, at pH 4, the arsenate species present in solution was H_2_AsO_4_^−^, and, at pH > 7.5, there was a predominance of the HAsO_4_^2−^ species. These three species are Lewis bases that can interact strongly with centre Fe^3+^ cations (Lewis acid). However, the negative charge on the surface of Nano-{Fe-BTC} at pH > 4 as a result of the deprotonation of the trimesic acid linkers (pKa_1_ = 2.86, pKa_2_ = 4.30, and pKa_3_ = 6.28) can promote repulsive forces with the anionic species such as H_2_AsO_4_^−^ and HAsO_4_^2−^, resulting in a decrease in the adsorption efficiency of As(V) by the Nano-{Fe-BTC} from 78% (pH = 2) to 50% (pH = 5) (Figure 4). On the other hand, the adsorption efficiency of As(V) by Basolite^®^F300 increased from 58% (pH = 2) to 95% (pH range 6.5 to 10.5) (Figure 4b). Taking into account that the chemical composition of both MOFs only differs in the percentage of iron (3.68% for the synthesised MOF and 2.98% for Basolite^®^F300), this difference may explain the highest efficiency of Nano-{Fe-BTC} at pH 2. The coordinative sites of arsenic species depend on the number of hydroxyl groups—three in the case of As(V)—which can offer four possible edge sites to form monodentate mononuclear complexes and six possible sites to form bidentate binuclear complexes with both Fe-BTC-based MOFs. The coordination between arsenate and the incompletely coordinated cationic Fe (Lewis acid) in the cluster through the formation of Fe−O−As bonds is the primary adsorption mechanism in iron-trimesate MOFs [34]. Moreover, the structural differences between these two MOFs also affected the adsorption efficiencies. The higher porosity (29.95%) and surface area (840 m^2^ g^−1^) of Basolite^®^F300 allowed more active sites for the electrostatic interaction between AsO_3_(OH)^2−^ and the centred Fe^3+^ despite the negatively charged surface of the MOF. Hence, the electrostatic interaction between arsenate and the positive charge of the centred Fe^3+^ enhances As(V) adsorption since the arsenate species present in solution were H_2_AsO_4_^−^ and HAsO_4_^2−^ at pH > 5.

Similar findings were obtained by the application of MIL-101(Fe), where the uptake of As(V) remained constant across the entire pH range (3–11) [32]. For MIL-53(Fe), an adsorption percentage of 99% was obtained at pH 5 and decreased to 87% at pH 11 [26]. The removal percentage of As(V) by Fe−BTC polymer was above 96% in the range of pH 2–10 and the maximum removal efficiency was 98.2% at pH 4, whereas, when the pH was increased to 12, the removal efficiency decreased to 35.8% as a result of the Fe-BTC instability in strong base conditions [29].

As for As(III), at a pH range of 2–8.5, the arsenite species present in solution was the neutral H_3_AsO_3_^−^ species, which explains the lower adsorption of As(III) (Figure S4). At pH values > 9, the predominance of the negatively charged H_2_AsO_3_^−^ species, which is considered to be a soft Lewis base, allows its interaction with the centred iron cations. As can be seen in Figure 4, As(III) adsorption percentages of 47% and 56% were obtained at equilibrium (pH 9.5) for Basolite^®^F300 and Nano-{Fe-BTC}, respectively. It should be noted, however, that As(III) adsorption by MIL-100(Fe) has also been reported to reach the maximum adsorption (120 mg g^−1^) at pH 7 [41]. To explain this result, these authors affirmed that As(III) binds in its neutral form (H_3_AsO_3_) with the Fe sites of the Fe-oxide layer, which we have not observed.

**Figure 5:**
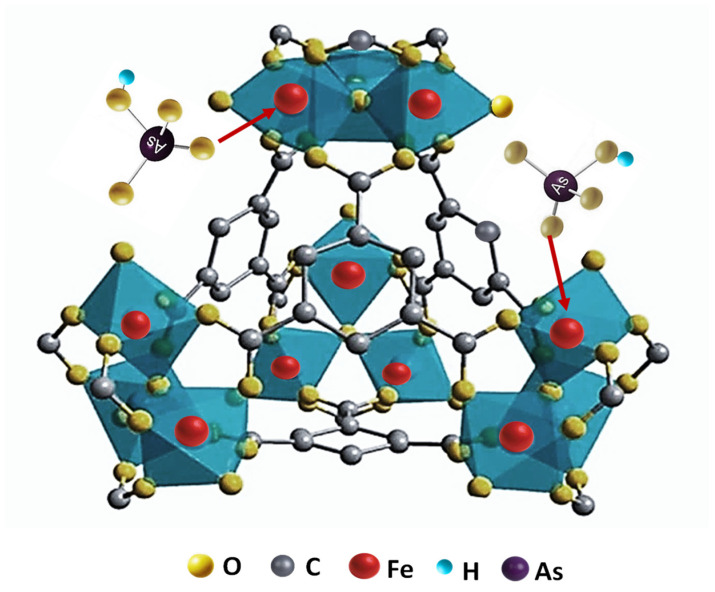
Schematic representation of the hybrid supertetrahedron of iron-trimesate MOFs built from iron(III) octahedral trimers and benzene-1,3,5-tricarboxylate moieties. The sites of interaction with arsenate species are also shown.

The possible adsorption mechanism in the case of Fe-BTC MOFs is illustrated in Figure 5, which is based on the iron-trimesate secondary building unit. The unsaturated sites of the Fe-BTC building unit are occupied by molecules of H_2_O that can be substituted by external molecules such as arsenate or arsenite to coordinate with Fe(III), as was described by the adsorption of arsenate by MIL-100(Fe) [27]. Arsenate is adsorbed through both surface-occurring and structure-occurring processes. The greater affinity of Basolite^®^F300 compared to Nano-{Fe-BTC} for As(V) has been attributed to the adsorption process as a result of the combination of factors where the attractive electrostatic forces are not dominant [42].

### 3.5. The Effect of Initial Arsenic Concentration

**Figure 6:**
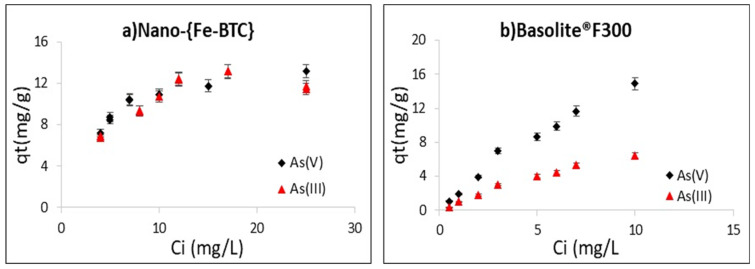
Effect of the initial arsenic concentration on the adsorption of As(III) and As(V) by: (**a**) 0.5 430 g L^−1^ Nano-{Fe-BTC}, pH_i_ = 11 for As(III), pH_i_ = 2 for As(V) and (**b**) 0.1 g L^−1^ of Basolite^®^F300 pH_i_ = 11 for 431 As(III), pH_i_ = 7 for As(V). t = 1 h and n = 2.

As can be seen in Figure 6a, the adsorption capacities of arsenite by Nano-{Fe-BTC} at pH 11 slightly increased to a maximum value of 13.12 mg g^−1^ for an As(III) concentration of 17 mg L^−1^, and, from this concentration onwards, there is a decrease in the adsorption capacity due to the lack of available sites on the surface of the adsorbent. The adsorption capacity at pH = 2 for As(V) increases until a concentration of 25 mg L^−1^ and the plot plateaus out at a maximum adsorption capacity of 13.5 mg g^−1^.

When Basolite^®^F300 was used as the adsorbent and the concentration of As(III) or As(V) was increased from 0.5 to 10 mg L^−1^, their adsorption capacities increased, indicating the high affinity of the arsenate and arsenite ions for this adsorbent. Saturation was not reached at the experimental conditions tested and it seems that Basolite^®^F300 is able to adsorb greater amounts of As(V) at pH = 6.5, as the relationship between the adsorption capacity and the initial concentration seems to be linear (Figure 6b). The adsorption capacities for As(III) of the commercial adsorbent are lower than for As(V) at pH 11. Moreover, a tendency can be observed to reach a plateau (saturation) with a maximum adsorption capacity of ≈6 mg g^−1^ (Figure 6b), which is lower than that obtained with Nano-{Fe-BTC} with the same experimental conditions.

### 3.6. The Effect of the Sorbent Concentration

The percentage of As(V) adsorbed by Nano-{Fe-BTC} from a 10 mg L^−1^ As(V) solution at pH 2 increased from 26.6% to 80.5% as the concentration of the sorbent was raised from 0.15 to 0.5 g L^−1^. On the other hand, when using Basolite^®^F300 with the experimental conditions of 10 mg L^−1^ As(V) and pH 6, the percentage of adsorbed As(V) was found to increase from 38% to 65%. These results may be explained by the higher iron content and increased available adsorption sites (higher pore size) of Nano-{Fe-BTC} in comparison with Basolite^®^F300. However, the percentage of adsorbed As(III) at pH 11 increased to a lesser extent, from 49% to 59%, when the sorbent concentration was raised from 0.25 g L^−1^ to 0.5 g L^−1^.

### 3.7. Adsorption Isotherms

**Figure 7:**
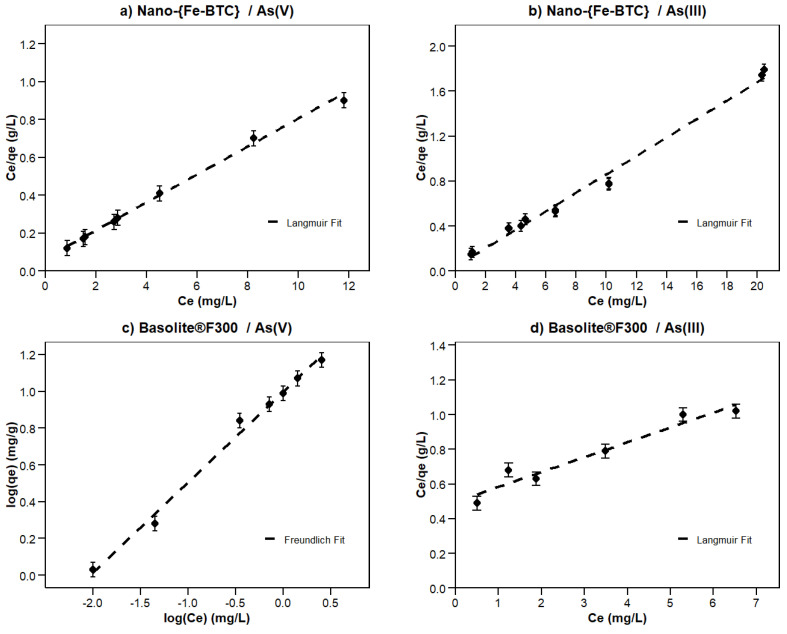
Fitting of the adsorption experimental points of As(III) and As(V) by Nano-{Fe-BTC} and Basolite^®^F300 as adsorbents to different adsorption isotherm models (linearised equations Table S2).

The fits of the experimental data to the Langmuir, Freundlich, Temkin, and Redlich–Peterson models were determined by using linearised equations (Table S2). The experimental points and the calculated models are depicted in Figure 7 and the parameters of the isotherm models are presented in Table 2. As the determination coefficients are relatively high for most of the models (R^2^ > 0.9), the comparison of these models based only on R^2^ is insufficient to select the model that best fits to the experimental data. Hence, the degree of agreement was assessed by also comparing the residual sum of squares of each model and by applying the Akaike’s information criteria (AIC).

The Langmuir and the Redlich–Peterson models provide the best fit with the highest R^2^ (Table 2). For the Redlich–Peterson model, the R^2^ were >0.96. except for the As(III) adsorption on Nano-{Fe-BTC}, whereas, for the Langmuir model, the lowest determination coefficients (R^2^ = 0.94) were found for As(III) adsorption by Nano-{Fe-BTC} and As(V) by Basolite^®^F300. The Langmuir empirical model assumes monolayer adsorption (the adsorbed layer is one molecule in thickness) in which adsorption can only occur at a finite (fixed) number of defined local sites, which are identical and equivalent, with no lateral interaction and steric hindrance between the adsorbed molecules even on adjacent sites. The surface coverage takes place by balancing the relative rates of adsorption and desorption (dynamic equilibrium). The Redlich–Peterson model considers the possibility of a multilayer mechanism in addition to the monolayer model (Langmuir). In order to elucidate which model performs best for the adsorption of As(V) by Nano-{Fe-BTC} and As(III) by Basolite^®^F300, a thorough comparison was made using AIC. This statistical test found that the Langmuir model outperforms the Redlich–Peterson model as it gave lower AIC scores, SS(AIC), in both cases. For the adsorption of As(III) by Nano-{Fe-BTC}, the Langmuir model gave the highest R^2^ 0.99 and it was concluded that this model gave the best fit to the experimental data.

For the adsorption of As(V) by Basolite^®^F300, the Freundlich model, which is associated with multilayer adsorption, exhibited a lower sum-of-square and higher R^2^ (R^2^ = 0.99) compared to the Langmuir model (R^2^ = 0.95). This result unequivocally indicates that the Freundlich model provides a superior fit to the experimental data, offering a more accurate representation of the underlying phenomena. The Redlich–Peterson model also provides a high R^2^ (0.96). However, based on the Akaike information criterion (AIC) comparison, we concluded that the Freundlich model was the most suitable option to explain the adsorption behaviour in our study. Moreover, the fact that the relationship between adsorption capacity and the initial concentration seems to be linear when increasing the initial concentration of As(V) from 0.5 to 10 mg L^−1^ (Figure 6b) supports the Freundlich model being the most appropriate for our experimental conditions.

## 4. Conclusions

In this study, we have compared the capacity of two iron-trimesate MOFs, synthesised Nano-{Fe-BTC} and commercial Basolite^®^F300, to adsorb inorganic arsenic species of As(III) and As(V). The results obtained showed that both MOFs can effectively adsorb inorganic arsenic species through chemisorption mechanisms. The efficiency of these iron-trimesate MOFs in adsorbing As(III) and As(V) depends significantly on the pH as they occur in different speciation conditions, as well as on the structural properties of both MOFs, which differ in porosity, pore size, and surface area. The properties of the MOFs also affected the kinetics of the adsorption processes. The maximum adsorption capacity of Nano-{Fe-BTC} for As(V) at pH 2 is mainly explained by the coordination between arsenate and the incomplete-coordinated cationic Fe (Lewis acid) in the cluster through the formation of Fe−O−As bonds [34], whereas, in the case of Basolite^®^F300, whose efficiency is greatest in the 6.6 to 10.5 pH range, the presence of anionic Lewis bases in the solution (H_2_AsO_4_^−^ and HAsO_4_^2−^) that interact strongly with centred Fe^3+^ cations (Lewis acid) seems to be the main adsorption process. In the case of As(III) at pHs > 9, the predominance of the negatively charged H_2_AsO_3_^−^ species, a soft Lewis base, allows its interaction with the centred iron cations. Higher arsenic levels were adsorbed by the commercial Basolite^®^F300 due to its greater surface area and porosity in comparison with Nano-{Fe-BTC}. Arsenate ions are found to have a greater affinity than arsenite ions for Fe-BTC materials.

## Figures and Tables

**Table 1:** Kinetic parameters and determination coefficients.

Model	Parameters	Basolite^®^ F300			Nano-{Fe-BTC}	
		(a) 50 rpm		(b) 20 rpm	(c) 20 rpm	
		As(V)	As(III)	As(V)	As(V)	As(III)
**PFO**	q_e_ (mg g^−1^)	21.827	13.996	4.943	12.359	1.832
	K_1_ (min^−1^)	0.044	0.037	0.090	0.053	0.025
	R^2^	0.936	0.962	0.969	0.981	0.336
**PSO**	qe (mg g^−1^)	41.667	22.222	12.195	14.925	10.000
	k_2_ (g mg^−1^ min^−1^)	0.005	0.001	0.075	0.007	0.156
	R^2^	0.996	0.904	0.999	0.974	0.999
**Elovich M1**	α (mg g^−1^ min^−1^)	85.426	1.889	271.792	7.142	19.427
	β (g mg^−1^)	0.195	0.266	0.749	0.541	0.483
	R^2^	0.980	0.948	0.992	0.962	0.987
**Elovich M2**	α’ (mg g^−1^ min^−1^)	3.26∙10^3^	-	1.44∙10^6^	6.55	-
	β’ (g mg^−1^)	0.29	−2.29∙10^14^	1.53	0.39	−3.83
	R^2^	0.94	-	0.97	0.97	0.71
**W-M M1**	KWM1 (mg g^−1^ min^−1/2^)	3.552	2.203	1.216	1.392	1.643
	DWM1	14.142	−1.812	6.334	2.209	4.122
	R^2^	0.998	0.945	0.972	0.978	0.985
**W-M M2**	KWM2 (mg g^−1^ min^−1/2^)	0.968	−1.02∙10^15^	0.230	0.696	−0.081
	DWM2	30.554	14.900	10.459	7.400	10.683
	R^2^	0.969	-	0.991	0.958	0.753
**LF-D M1**	D	0.040	0.037	0.112	0.036	0.202
	R^2^	1.000	0.907	0.998	0.933	0.975
**LF-D M2**	D	0.014	-	0.036	0.061	−0.030
	R^2^	1.000	-	1.000	0.988	0.920

**Table 2:** Isotherm parameters, determination coefficients, and scores of the Akaike’s information criteria (SS(AIC)).

		Nano-{Fe-BTC}		Basolite^®^F300	
Model	Parameters	As(V)	As(III)	As(V)	As(III)
**Langmuir**	q_m_ (mg g^−1^)	13.61	12.20	16.16	11.72
	K_L_ (L mg^−1^)	1.05	1.99	2.38	0.17
	R^2^	0.99	0.99	0.95	0.94
	SS(AIC)	0.64	3.23	-	0.21
**Freundlich**	n	4.84	4.76	2.02	1.39
	K_F_ (L g^−1^)	7.90	7.29	10.12	1.70
	R^2^	0.94	0.76	0.99	0.99
	SS(AIC)	-	-	1.12	-
**Temkin**	K_T_ (L mg^−1^)	44.76	39.82	80.65	2.52
	B (mg g^−1^)	2.05	1.98	2.40	2.10
	R^2^	0.96	0.73	0.93	0.95
**Redlich–Peterson**	βRP	0.79	0.79	0.51	0.28
	K_RP_ (L g^−1^)	7.90	7.29	10.01	1.70
	R^2^	0.98	0.90	0.96	0.98
	SS(AIC)	4.07	6.29	6.19	0.37

## Data Availability

The original contributions presented in this study are included in the article/Supplementary Material. Further inquiries can be directed to the corresponding author.

## Supplementary Materials

The following supporting information can be downloaded at: https://www.mdpi.com/article/10.3390/nano15010036/s1.
