# Supplementary material for: New Insights on Iron-Trimesate MOFs for Inorganic As(III) and As(V) Adsorption from Aqueous Media
Source: Nanomaterials (Basel). 2024 Dec 29;15(1):36. doi: 10.3390/nano15010036 (PMC11723219; doi:10.3390/nano15010036)
Supplement: Supplementary file 1 [file nanomaterials-15-00036-s001.zip › nanomaterials-3325372-supplementary.pdf]

Supplementary materials:

Table S1. Kinetic models: linear equations and corresponding parameters.

Kinetic model	Linear equation	Parameter
Pseudo-first-order	$\log(q_e - q_t) = \log q_e - \frac{K_1}{2.303} t$	$q_e\ (mg\ g^{-1}) = adsorption\ capacity\ at\ equilibrium$<br>$q_t\ (mg\ g^{-1}) = adsorption\ capacity\ at\ time\ t$<br>$K_1\ (L\ min^{-1}) = first - order\ adsorption\ rate\ constant$
Pseudo-second-order	$\frac{t}{q_t} = -\frac{1}{K_2\ q_e^2} + \frac{1}{q_e}\ t$	$K_2\ \ (g\ mg^{-1}\ min^{-1}) = second - order\ adsorption\ rate\ constant$
Elovich	$q_t = \frac{1}{\beta} Ln(\alpha\beta) + \frac{1}{\beta} ln(t)$	$\alpha\ \ (mg\ g^{-1}\ min^{-1}) = Elovich\ initial\ adsorption\ rate$<br>$\beta\ \ (g\ mg^{-1})\ = desorption\ constant$
Weber–Morris	$q_t = K_{WM}\ t^{0.5} + D_{WM}$	$K_{WM}\ \left(mg\ g^{-1}\ min^{-\frac{1}{2}}\right) = Weber - Morris\ constant$<br>$D_{WM}(mg\ g^{-1})\ = constant\ of\ any\ experiment$
Liquid film diffusion	$Ln\left(1 - \frac{q_t}{q_e}\right) = -D_{LFD}\ t$	D_{LFD}

Table S2. Isotherm models: linear equations and corresponding parameters.

Kinetic model	Linear equation	Parameter
Langmuir	$\frac{C_e}{q_e} = \frac{1}{q_{max} k_l} + \frac{1}{q_{max}} C_e$	$q_e\ (mg\ g^{-1}) = adsorption\ capacity\ at\ equilibrium$<br>$q_{max}\ (mg\ g^{-1}) = maximum\ adsorption\ capacity\ at\ time\ t$<br>$K_l\ (L\ mg^{-1}) = adsorption - related\ constant$
Freundlich	$q_e = K_f C_e^{1/n}$	$q_e\ (mg\ g^{-1}) = adsorption\ capacity\ at\ equilibrium$<br>$K_f\ = adsorption\ intensity$<br>$n\ = adsorption\ capacity$
Temkin	$q_e = BLn(A) + B\ ln(C)$	$A\ \ (L\ mg^{-1}) = Temkin\ constant$<br>$B\ (J\ mol^{-1})\ = Temkin\ constant$
Redlich–Peterson	$log\left(\frac{C_e}{q_e}\right) = log\ k_R + \beta_R T Ln(C_e)$	$K_{Rp}\ (g\ L^{-1})\ = Redlich - Peterson\ constant$<br>$\beta_{Rp} = desorption\ constant$

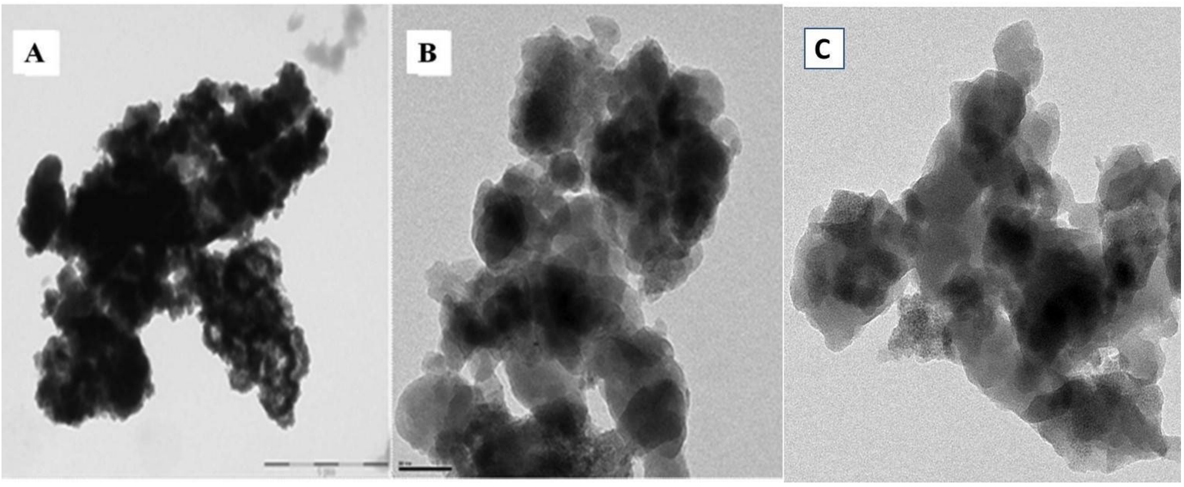

Figure S1. TEM images of A) Basolite®F300 and Nano-{Fe-BTC}: B) 20 nm and C) 50 nm.

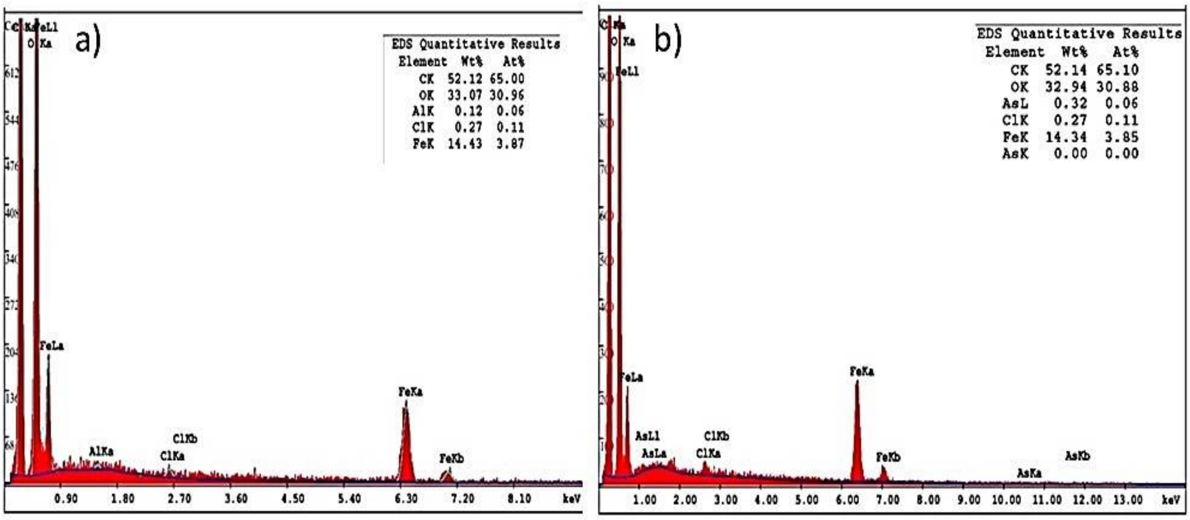

Figure S2. EDS spectra of Nano-{Fe-BTC} before (**a**) and after adsorption of As(V) (**b**) showing their corresponding elemental compositions.

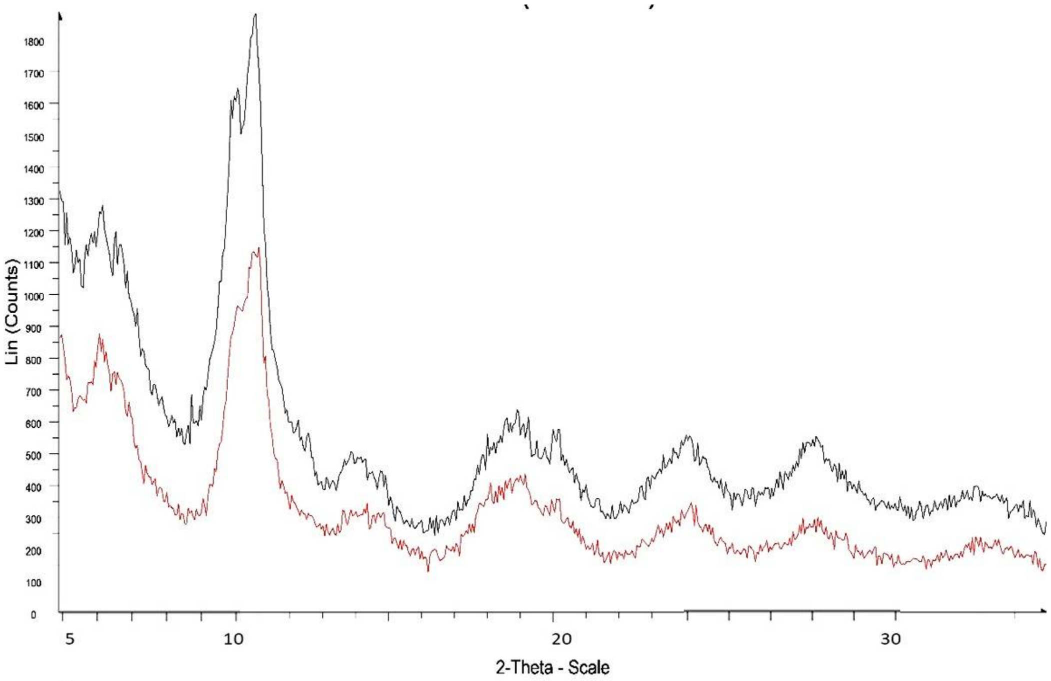

Figure S3. XRD spectra of Basolite®F300 before the adsorption of As(V) (red) and after the adsorp tion of As(V) (black).

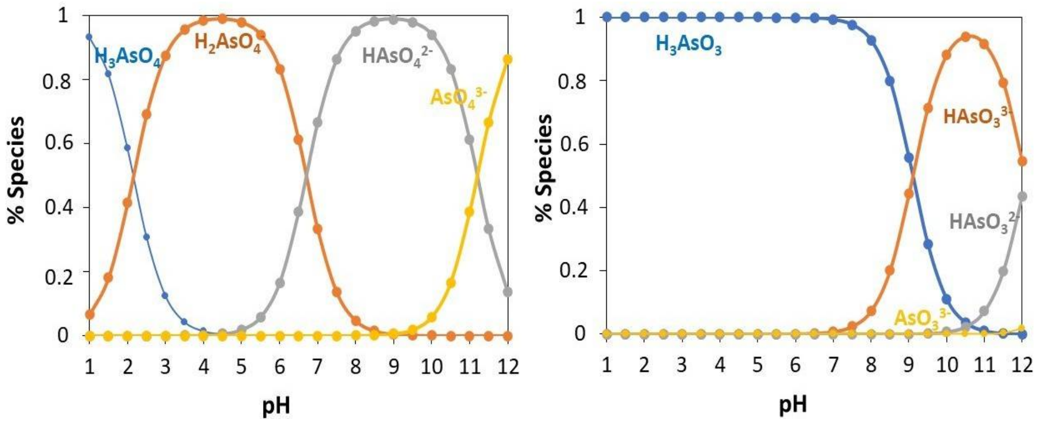

Figure S4. Speciation diagrams of As(III) and As(V).

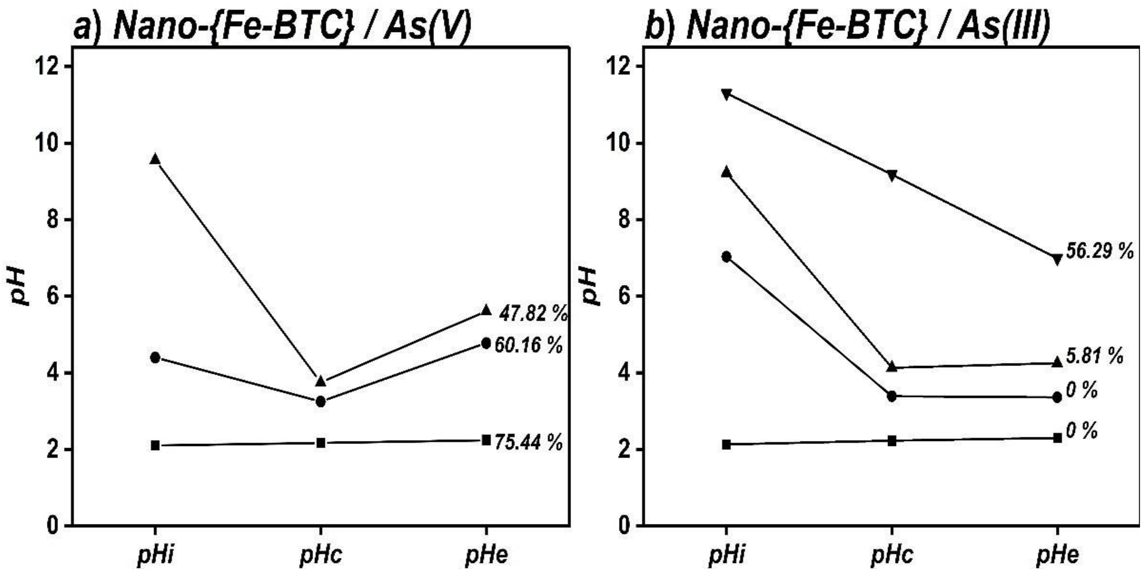

Figure S5. Adsorption capacity percentage at the equilibrium and pH variation after the addition of 10 mg of Nano-{FeBTC} to 20 mL of 10 mg L^{-1} of a) As(V) and b) As(III), t=1h at 20 rpm**.** pH_i : initial pH of the As solutions, pH_c: pH after the addition of the Fe-BTC MOF to the As solution, and pH_e: pH at the equilibrium. .
